# Postpartum Morbidity Associated With Advanced HIV Disease

**DOI:** 10.1155/IDOG/2006/79512

**Published:** 2006-11-19

**Authors:** Judette Louis, Mudathiru A. Buhari, Dianne Allen, Bernard Gonik, Theodore B. Jones

**Affiliations:** ^1^Department of Obstetrics and Gynecology, Division of Maternal Fetal Medicine, Wayne State University, Detroit, MI 48201, USA; ^2^Department of Internal Medicine, Division of Infectious Diseases, Wayne State University, Detroit, MI 48201, USA

## Abstract

*Objective*. To investigate the postpartum morbidity and postpartum management of febrile morbidity associated with advanced HIV infection. *Methods*. A case control study of HIV infected women at a tertiary care center during January 2000–June 2005 was performed. Postpartum morbidity was defined as endometritis, blood transfusion, wound complication, readmission, infectious morbidity, or unexpected surgery. *Results*. Women in Group 1 had AIDS (*N* = 33), Group 2 were relatively immunocompetent HIV infected women (*N* = 115), and Group 3 were uninfected women (*N* = 152). Group 1 was more likely to have a postpartum morbidity (32.3 versus 19.3 and 13.2%, *P* = .03) and to have postpartum imaging 18.8 versus 7.9 and 2.6%, *P* = .002. After controlling for potential confounders, cesarean delivery (OR 6.2, 95% CI 2.1–505.5) but not advanced HIV disease was associated with an increased risk of postpartum morbidity. *Conclusion*. Cesarean delivery and not advanced HIV disease increases the risk of postpartum morbidity in women with AIDS.

## INTRODUCTION

Women are making up an increasing part of the acquired immune deficiency syndrome (AIDS) epidemic in the United States [1]. In 2001 HIV infection was the 6th leading cause of death among all women aged 25–34 years and the 4th leading cause of death among all women aged 35–44 years. By the end of 2003, women and adolescents represented 22% of the people living with AIDS [1]. With the considerable number of women of childbearing age with AIDS, issues related to pregnancy remain a concern. Initial studies in the field of perinatal HIV focused on vertical transmission. As we have made great strides to reduce vertical transmission to less than 2% in the US, there has been an increased focus on maternal health.

While childbirth in developed countries is relatively safe, there exists a risk of morbidity. Of significance, cesarean section is associated with an increased risk and, in particular, infectious morbidity [2]. Risk factors for morbidity include prolonged rupture of membranes, obesity, immune suppression, and type of anesthesia, nonelective cesarean delivery, and a number of vaginal examinations among others [3]. Women with AIDS are severely immunocompromised and may be at increased risk for postpartum morbidity. While studies have indicated that the risk may be greater for HIV-infected patients, few studies have estimated the risk among the most immunocompromised of these patients [4]. Further more, no studies have examined the difference in postpartum management. This study sought to estimate the risk of postpartum morbidity among patients with AIDS compared to more immunocompetent HIV-infected patients and HIV-uninfected women. An additional objective was to determine the impact of their diagnosis on the management of puerperal infections.

## SUBJECTS AND METHODS

This retrospective chart review was approved by the Institutional Review Board at Wayne State University. All of the patients delivered at a single urban tertiary care center. Cases consisted of patients with a diagnosis of HIV infection who delivered from January 1, 2000–June 1, 2005. Uninfected controls were selected utilizing a computerized perinatal database and random number allocation. Charts with incomplete medical records or undocumented HIV testing during the current pregnancy were excluded. Medical charts were reviewed for demographic data, clinical characteristics, antenatal course, delivery data, and postpartum management. Among HIV-infected patients, treatment data were recorded including CD4 cell count, HIV-1 RNA viral load measurements, and use of highly active antiretroviral therapy (HAART). Group 1 consisted of patients diagnosed with AIDS as defined by a current or prior history of a CD4 cell count < 200 cells/*μ*L or an opportunistic infection [5]. Group 2 consisted of relatively immunocompetent HIV-infected patients (CD4 ≥ 200 cells/*μ*L) and Group 3 consisted of HIV-uninfected patients. Febrile morbidity was defined as a temperature ≥ 38.0°C 24 hours or greater postdelivery. Postpartum endometritis was defined as persistently elevated temperature with uterine tenderness in the absence of evidence of a nonuterine etiology. Postpartum morbidity was defined as anemia requiring a blood transfusion, endometritis, other infectious morbidity, wound complication, repeat admission for a pregnancy-related complication within 6 weeks postdelivery, or unexpected surgery. Infectious morbidity was defined as the presence of endometritis, wound infection, or pneumonia. Data regarding the indication for use of intravenous antibiotics, the use of imaging in assessment of febrile morbidity, and the time from the first fever until initiation of intravenous (IV) antibiotics were collected as well. Postdelivery length of stay (LOS) was defined as the time from delivery until discharge from the hospital.

## STATISTICAL ANALYSIS

Statistical analysis was performed utilizing the SPSS statistical software (version 14.0 SPSS Inc, Chicago, IL). Wilcoxon ranks sum test and the Kruskal-Wallis test were utilized for discrete and continuous variables. The chi square test was utilized for binomial variables. A logistic regression was performed to evaluate possible confounders. Postpartum morbidity was the outcome of interest. The variables included were government insurance, age, body mass index (BMI), nulliparity, drug abuse history, smoking, cesarean section after labor, intrapartum fever, general anesthesia, duration of membrane rupture, and cesarean delivery. A probability of *P* < .05 was significant.

## RESULTS

There were 148 HIV-infected patients with complete data. Of that group, 33 were diagnosed with AIDS by CDC criteria and 7 of those women had symptomatic AIDS. The uninfected control group consisted of 152 HIV-uninfected women. Table 1 consists of demographic data by group. HIV-infected women were more likely to smoke (24.2 and 28.9 versus 15.8%, *P* = .03), have a drug abuse history (33.3 and 24.6 versus 5.3%, *P* < .0001), were older (median 28 and 28 versus 24 years, *P* < .0001), and had a lower BMI (median 27 and 27 versus 32 kg/m^2^, *P* < .0001). Overall, 82% of the HIV-infected women were adherent with their antretroviral therapy. Clinical data are presented in Table 2. Women with AIDS were more likely to have a cesarean delivery (59.4 versus 35.1 and 15.8%, *P* < .0001) and there was no difference in the use of prophylactic antibiotics at the time of cesarean section. Among women with a vaginal delivery, patients with AIDS had a shorter duration of rupture of membranes (median 50 minutes versus 63 and 193 minutes, *P* < .0001) but there was no difference in the number of vaginal exams or the duration of labor. Women with AIDS were more likely to have a postpartum morbidity (32.3 versus 19.3 and 13.2%, *P* = .03) or an infectious morbidity (22.6 versus 11.54 and 5.3%, *P* = .007). The highest rate of infectious morbidity was among women with symptomatic AIDS (66%). There was no difference in febrile morbidity, hemorrhage, wound complications, and multiple complications among the three groups. Women with AIDS were more likely to have radiological imaging in the evaluation of febrile morbidity and a resultant delay in initiation of IV antibiotics but there was no difference in the rate of antibiotic use (Table 3). Among the imaging modalities, chest radiography was performed in 13 of the 19 patients who had imaging. One demonstrated pneumonia and the rest were negative. Abdominal and pelvic computed axial tomography was performed in 4 of the 19 of the patients, yielding the diagnosis of a pelvic abscess in one patient and a small bowel obstruction in another. Two patients had a head and neck magnetic resonance imaging (MRI); one demonstrated lesions suggestive of cryptococcal meningitis. In a logistic regression controlling for potential confounders, cesarean delivery (OR 19.5, 95% CI 5.4–69.8) and not advanced HIV disease were associated with an increased risk of postpartum morbidity (see Table 4).

## DISCUSSION

We found an association between advanced HIV disease and postpartum morbidity. In particular, infectious morbidity was diagnosed more frequently in women with advanced HIV disease than in relatively immunocompetent HIV-infected women and in HIV-uninfected women. When controlling for potential confounders, the majority of the risk appeared to be associated with cesarean delivery and not HIV immune status. Additionally, in the management of postpartum febrile morbidity, there was an increased use of imaging and a delay in initiation of antibiotics among women with AIDS.

Our finding is similar to a previously published study by Marcollet et al. In a retrospective review of 401 HIV-infected women, Marcollet et al found that women with AIDS were more likely to have febrile morbidity but there was no difference in postpartum complications [6]. In that study, the predominant risk was associated with cesarean delivery. Watts et al in a study of 497 women participating in the Pediatric Aids Clinical Trial Group study of HIV-infected women with a CD4 count below 500 cells/*μ*L demonstrated an increased risk of postpartum morbidity but that risk was associated with cesarean delivery [7]. Other studies that estimated risk focused only on patients undergoing cesarean delivery. In those studies, risk appeared to be associated with the degree of immune suppression. Semprini et al in a study of 156 HIV-infected women undergoing cesarean delivery and controlling for potential confounding variables demonstrated that profound immunodeficiency (defined as a CD4 count below 200 cells/*μ*L) was the predominant risk factor for postpartum morbidity [8]. Similarly, Maiques-Montesinos et al in a case control study of HIV-infected women undergoing cesarean section demonstrated that the lowest risk of morbidity was among HIV-infected women with a CD4 count > 500 cells/*μ*L compared to more immunosuppressed patients [9]. The obvious inconsistencies in the findings among the different studies cited may be related to differences in sample size and patient population.

In our study, we found an increased use of imaging in the evaluation of febrile morbidity among women with AIDS and a resultant delay in initiation of antibiotics. There was no difference in the overall use of IV antibiotics. The management of febrile morbidity among these immunosuppressed women is a question that has yet to be answered. In the uninfected population, prophylactic antibiotics have been demonstrated to decrease perioperative morbidity associated with cesarean delivery [2, 10]. Longer duration of antimicrobial therapy has not been advocated. However, those studies did not include the evaluation of HIV-infected women. Our data indicate that interventions for decreasing postcesarean infectious morbidity in women with AIDS warrant investigation.

In our cohort, the predominant indication for elective cesarean delivery was a high viral load (data not shown). In a randomized trial, the European Mode of Delivery Collaborative demonstrated a 50% reduction of vertical transmission with the use of scheduled cesarean section [11]. This finding was confirmed by a large meta-analysis [12]. The findings of these studies resulted in the release of a committee opinion by the American College of Obstetricians and Gynecologists, which indicated that patients with a viral load > 1000 copies/ml should be counseled and offered an elective cesarean section at 38 weeks of gestation to decrease the risk of vertical transmission [13]. Since that time, the use of cesarean section as a tool in the prevention of maternal-to-child transmission has increased [14]. While this has been an effective part of the management to decrease perinatal transmission, it does so at a maternal cost. Among high-risk patients who may present late for prenatal care, prompt diagnosis and initiation of highly active retroviral treatment play a crucial role. In particular, women with advanced HIV infection may have a higher baseline viral load or therapeutic resistance requiring therapy manipulation [15]. Effective therapy may decrease the number of women requiring a cesarean delivery thus decreasing the associated perioperative morbidity.

This study is limited in that it is a retrospective review at a single institution. The patient population was particularly in high risk with regards to entry to care and substance abuse. Providers were not blinded in regards to immune status. As such, that information may have impacted their management decisions. Additionally, the small-sample sizes may preclude us from detecting an association that may truly exist. Regardless, this study illustrates that women with AIDS face additional risks with cesarean delivery. These findings would confer an additional duty for the perinatal care provider to include more information about these risks in the extensive counseling given to women with AIDS when planning for mode of delivery prior to initiation of labor. These findings emphasize further the importance of early initiation of prenatal care to allow time for viral load suppression and place HIV care providers for women of childbearing age at the leading edge of this most important care initiative.

## Figures and Tables

**Table 1:** Demographic data by group.^†^

	Group 1 (AIDS)	Group 2 (HIV infected)	Group 3 (HIV uninfected)	*P*-value*
	*N* = 33	*N* = 115	*N* = 152	

Black	27(81.8)	102(89.5)	128(84.2)	.36
Caucasian	2(6.1)	8(7.0)	12(7.9)	.92
Hispanic	4(12.1)	2(1.8)	12(7.9)	.03
Insurance government	24(72.7)	73(65.2)	128 (84.2)	.002
Tobacco	8(24.2)	33(28.9)	24(15.8)	.03
Drug use	11(33.3)	28(24.6)	8(5.3)	< .0001
Age (years)	28(24–32)	28(23–35)	24(19–28)	< .0001
BMI (kg/m^2^)	27(22–32)	27(24–35)	32(28–37)	< .0001
Adherent antiretroviral use	28(84.8)	93(80.9)	—	N/A
Predelivery CD4 (cells/mm^3^)	185(108–310)	534(420–720)	—	N/A

^†^Data presented as N (%) or median (interquartile range).

*Represents comparison across groups.

**Table 2:** Clinical data by group.^†^

	Group 1 (AIDS)	Group 2 (HIV infected)	Group 3 (HIV uninfected)	*P*-value*
	*N* = 33	*N* = 115	*N* = 152	

Cesarean section	19(59.4)	40(35.1)	24(15.8)	< .0001
Elective	15(46.9)	23(20.2)	4(2.7)	< .0001
Emergent	3(9.4)	9(7.9)	8(5.3)	.57
General anesthesia	1(3.7)	6(5.4)	1(.7)	.06
Duration of labor (hours)**	6(3–8)	9.5(6–13)	8.3(5.6–11)	.07
Duration of membrane	50 (5.5–390.5)	63(9–171.5)	192 (96–441.5)	< .0001
rupture (minutes)**				
Vaginal exams**	3(3–6)	4(3–6)	4(3–5)	.07
Febrile morbidity	6(18.8)	15(13.2)	24(15.8)	.70
Any complication	10(32.3)	22(19.3)	20(13.2)	.03
Multiple complications	3(9.7)	7(6.1)	4(2.6)	.16
Infectious morbidity	7(22.6)	13(11.5)	8(5.3)	.007
Wound complication	1(3.2)	6(5.3)	8(5.3)	.89
Hemorrhage	0	2(1.8)	8(5.3)	.16

^†^Data presented as *N* (%) or median (interquartile range).

*Represents comparison across groups.

**Among women with vaginal delivery.

**Table 3:** Management of febrile morbidity.^†^

	Group 1	Group 2	Group 3	*P*-value*

Duration of fever (d)	3(1–6)	3(1–4)	2(1–4)	.54
Imaging	6(18.8)	9(7.9)	4(2.6)	.001
IV antibiotics	5(83.3)	12(80.0)	16(66.7)	.54
Time to ABX initiation (h)	9 (3–27)	7(1–57)	1(1–8)	.015
LOS (d)	6(2–8)	4(3–5)	4(2–5)	.39

^†^Data presented as *N* (%) or median (interquartile range).

*Represents comparison across groups.

**Table 4:** Logistic regression modeling for risk factors for postpartum morbidity.

	Odds ratio	95.0% CI

Group 1	.66	.14–3.1
Group 2	.673	.19–2.4
Government insured	1.214	.36–4.1
Age	.959	.864–1.1
Nulliparous	1.161	.34–3.9
BMI	.984	.9–1.04
Drug abuse history	.640	.14–2.9
Smoking	1.647	.5–5.3
Cesarean after labor	.686	.1–4.7
Intrapartum fever	.087	.009–.84
Duration of rupture of membranes	1.002	1.0–1.004
Cesarean delivery	19.486	5.4–69.8

## References

[1] CDC HIV/AIDS Surveillance Report.

[2] Nielsen TF, Hokegard KH (1983). Postoperative cesarean section morbidity: a prospective study. *American Journal of Obstetrics and Gynecology*.

[3] Yonkers M (1985). Risk factors for postcesarean endomyometritis. *The American Journal of Medicine*.

[4] Panburana P, Phaupradit W, Tantisirin O, Sriintravanit N, Buamuenvai J (2003). Maternal complications after caesarean section in HIV-infected pregnant women. *Australian and New Zealand Journal of Obstetrics and Gynaecology*.

[5] Bartlett JG, Gallant JE (2005). *Medical Management of HIV Infection*.

[6] Marcollet A, Goffinet F, Firtion G (2002). Differences in postpartum morbidity in women who are infected with the human immunodeficiency virus after elective cesarean delivery, emergency cesarean delivery, or vaginal delivery. *American Journal of Obstetrics and Gynecology*.

[7] Watts DH, Lambert JS, Stiehm ER (2000). Complications according to mode of delivery among human immunodeficiency virus-infected women with CD4 lymphocyte counts of ≤ 500/*μ*L. *American Journal of Obstetrics and Gynecology*.

[8] Semprini AE, Castagna C, Ravizza M (1995). The incidence of complications after caesarean section in 156 HIV-positive women. *AIDS*.

[9] Maiques-Montesinos V, Cervera-Sanchez J, Bellver-Pradas J, Abad-Carrascosa A, Serra-Serra V (1999). Post-cesarean section morbidity in HIV-positive women. *Acta Obstetricia et Gynecologica Scandinavica*.

[10] ACOG (2003). ACOG practice bulletin number 47, October 2003: prophylactic antibiotics in Labor and delivery. *Obstetrics and Gynecology*.

[11] Parazzini F, Ricci E, Di Cintio E (1999). Elective caesarean-section versus vaginal delivery in prevention of vertical HIV-1 transmission: a randomised clinical trial. European Mode of Delivery Collaboration. *Lancet*.

[12] The International Perinatal HIVG (1999). The mode of delivery and the risk of vertical transmission of human immunodeficiency virus type 1: a meta-analysis of 15 prospective cohort studies. *New England Journal of Medicine*.

[13] ACOG (2001). ACOG committee opinion scheduled cesarean delivery and the prevention of vertical transmission of HIV infection. *International Journal of Gynecology & Obstetrics*.

[14] Dominguez KL, Lindegren ML, D'Almada PJ (2003). Increasing trend of cesarean deliveries in HIV-infected women in the United States from 1994 to 2000. *Journal of Acquired Immune Deficiency Syndromes*.

[15] Bartlett JGGJE (2005). *Medical Management of HIV Infection*.

